# Whole genome sequence of *Mycoplasma ovipneumoniae* strain NXNK2203 isolated from Hu sheep in Ningxia province, China

**DOI:** 10.1128/MRA.00565-23

**Published:** 2023-07-24

**Authors:** Jiandong Wang, Hongyan Liu, Abdul Raheem, Qing Ma, Xiaojun Liang, Yanan Guo, Doukun Lu

**Affiliations:** 1 NingXia Academy of Agricultural and Forestry Sciences, Yinchuan, China; 2 School of Agriculture, Ningxia University, Yinchuan, China; 3 National Key Laboratory of Agricultural Microbiology, Huazhong Agricultural University, Wuhan, China; University of Rochester School of Medicine and Dentistry, Rochester, New York, USA

**Keywords:** whole genome sequence, *Mycoplasma ovipneumoniae*

## Abstract

We report whole-genome sequencing of *Mycoplasma ovipneumoniae* strain NXNK2203 isolated from the lungs of dead Hu sheep in Ningxia province, China. We get single circular chromosomes of 1,014,835 bp after sequencing. The purpose was to better understand the clinical significance of *Mycoplasma* infections for the health and welfare of ovine species.

## ANNOUNCEMENT

Throughout the world, *Mycoplasma ovipneumoniae* is an important cause of sheep respiratory disease, which is a major disorder in livestock animals (1 - 3).

To isolate bacteria, samples of pneumonic lungs were collected via biopsy from *Ovis aries* (Hu sheep) in Ningxia province, China. The collected samples were inoculated on pleuropneumonia-like organisms (PPLO) agar plates (kept at 37°C, 5% CO2, and 98% humidity). The isolate was initially identified using conventional methods (observing “fried-egg” colonies) on PPLO agar plates, followed by 16s RNA sequencing. To gain a comprehensive understanding of the bacterial characteristics, whole genome sequencing was performed by PacBio sequencing technology using a molecule-real-time technology (4) (SMRT) chip as the sequencing carrier after checking its integrity by gel electrophoresis (5). The DNA was sheared using g-TUBE (Covaris), and the fragmented DNA underwent nick and end repairing. MRTbell adapters were ligated to the prepared DNA fragments. An exonuclease treatment was applied to degrade failed ligation products. Fragments of the desired size were selected using BluePippin and prepared for sequencing on the Sequel II system with a sequencing depth of >100×. The SMRTbell Express Template Prep Kit (PacBio) was used for library preparation. Then Lima (version 2.0.0) software was employed for demultiplexing and adapter removal from SMRT sequencing data (6). Read quality control and length filtering were performed using SMRT Link Suite (version 10.1.0) (7). Error correction was carried out using Pilon (version 1.24) and Canu (version 2.1.1) software (8, 9). GS *De Novo* Assembler v. 2022 was utilized for assembly, resulting in contiguous sequences (contigs). A total of 859,750,776 reads were obtained after filtering short-length circular consensus sequencing reads, their *de novo* assembly, and error correction of the assembled genome. The maximum read length was 44,767 with a mean length of 7,441, and the mean quality score was 42.36. After further filtering the reads with shorter lengths and retaining the reads with a length of more than 2 kb, a total of 850,842,470 reads with a mean read length of 7,739 bp, a maximum read length of 44,767, and a mean quality score of 41.51 were obtained. *M. ovipneumoniae* strain NXNK2203 consists of single circular chromosomes, which were assessed by Circlator v1.5.5. Circlator v1.5.5 analyzes read alignments, clusters overlapping reads, closes gaps, and assesses continuity to determine circularity, ultimately reconstructing circular chromosomes from genome assemblies (10) with 1,014,835 bp and 29.23% GC content. A total of 686 genes with minimum lengths of 114 bp, maximum lengths of 12,057 bp, and average lengths of 1,279 bp were obtained that were predicted by Prodigal v2.6.3 (11). The RepeatMasker v4.0.5 (12) was used to predict the repetitive sequence length and content, which were found to be 12,502 bp and 1.23%, respectively. The genome encodes 3 rRNA and 30 tRNA, predicted by Infernal v1.1.3 (13) and tRNAscan-SE v2.0 (14), respectively. A total of six clustered regularly interspaced palindromic repeats were found by CRT v1.2 (15). The genome also has four prophages, predicted by PhiSpy v2.3 (16). Default parameters were used for all software.

## Data Availability

This whole genome sequence was deposited at NCBI GenBank under accession number CP124621, and raw data was submitted in NCBI sequence read archive under accession number SRR24462972 with BioProject number PRJNA963935. The publicly available annotation (NCBI) was completed using Prokaryotic Genome Annotation Pipeline (PGAP). The annotation data described in the manuscript corresponds with the reported PGAP annotation.
